# #Bartender: portrayals of popular alcohol influencer’s videos on TikTok^©^

**DOI:** 10.1186/s12889-024-18571-1

**Published:** 2024-05-23

**Authors:** Erell Guégan, Marco Zenone, Mélissa Mialon, Karine Gallopel-Morvan

**Affiliations:** 1 Univ Rennes, EHESP, CNRS, Inserm, Arènes - UMR 6051, RSMS (Recherche sur les Services et Management en Santé) - U 1309, Rennes, France; 2 Faculty of Public Health & Policy, London School of Hygiene and Tropical Medicine, London, UK; 3 Department of Occupational Science and Occupational Therapy, University of British Columbia, Vancouver, Canada; 4 Trinity Business School, Trinity College Dublin, Dublin, Ireland

**Keywords:** Marketing, Online, Digital, Social media, Alcohol

## Abstract

**Background:**

Despite widespread use of the short-video social media platform TikTok**©**, limited research investigates how alcohol is portrayed on the platform. Previous research suggests that a driver of alcohol content on TikTok**©**, in part, comes from bartenders demonstrating how to make drinks. This study aims to explore the characterizing patterns of how bartender influencers on TikTok**©** feature and incorporate alcohol in their videos.

**Methods:**

We identified the global top 15 most followed bartenders on TikTok**©** in 2021 (cumulative 29.7 million subscribers) and the videos they posted in November and December 2021, the period just before Christmas and New Year, when alcohol tends to be more marketed than in other periods. The videos were coded based on five criteria: (1) the presence of alcohol or not; (2) alcohol categories; (3); alcohol brand(s) if visible; (4) the presence of candies and other sweet products; (5) presence of cues that refer to young people’s interests.

**Results:**

In total, we identified 345 videos, which received 270,325,600 views in total, with an average of 18,021,707 views per video. Among these 345 videos, 92% (n = 317) displayed alcohol in their cocktail recipes (249,275,600 views, with an average of 786,358 views). The most common types of alcohol present in videos were liquor, vodka, rum, and whiskey, all of which are high-ABV beverages. 73% (n = 230) displayed or mentioned an alcohol brand. 17% (n = 55) associated alcohol with sweet products such as different types of candy (53,957,900 views, with an average of 981,053 views per video). 13% (n = 43) contained cues appealing to young people (e.g., cartoons, characters) (15,763,300 views, with an average of 366,588 views per video).

**Conclusions:**

Our findings suggest a large presence of positively framed alcohol content posted by popular bartenders on TikTok**©**. As exposure to digital marketing is related to an increase in alcohol consumption, particularly among young people, regulations are needed to protect the public from alcohol-related harms.

## Background

Alcohol consumption is a public health concern, particularly among young people. High alcohol consumption increases drunk driving, physical altercations, sexual assault, and the use of illicit drugs, and also has detrimental effects on brain development, neuropsychological performance and increases the risk of becoming alcohol dependent [1]. Worldwide, according to the World Health Organization (WHO, 2022), more than a quarter of 15–19 year old reported drinking alcohol in 2016, amounting to 155 million adolescents, and the prevalence of heavy episodic drinking among them was 13.6%, although that consumption is declining [2].

The influence of digital marketing by alcohol brands is a significant contributing factor to high alcohol consumption in young people and other segments of the population [3–5]. In the world, 4.76 billion people are social media users [6], a large market for the alcohol industry to target, and among them, 12% of users are aged 13–19, and 31% are aged 20–29 [7]. Young people are the largest group of TikTok users, with those aged 13–17 representing a third of all users on the platform [8].

Digital messages promoting alcohol come in various forms: paid targeted social media ads, official brand accounts (websites), the partnership of events (i.e. concerts, sporting events), popular influencers paid by alcohol companies, or user-generated posts [9–13]. Our study investigates the promotion of alcohol by social media influencers. Influencer marketing is a noted effective practice because influencers build strong relationships of trust and confidence with their followers, who view their content as providing valuable information and advice [14].

Limited research has analyzed how digital influencers portray alcohol products. Hendriks et al. [15] revealed that 63% of content created by Instagram influencers had at least one post related to alcohol, and all these posts were positive: they reinforce the “fun” image of alcohol and normalize it. Focusing on the 100 most popular videos of TikTok**©** alcohol-related user-generated content, Russell et al. [16] showed positive content (humour and camaraderie) and the promotion of rapid alcohol consumption. Vranken et al. [17] noted that young people are frequently exposed to Instagram influencers who hold alcoholic beverages, provide positive reviews for brands, and promote their own beverages. Young people in that study reported enjoying images of influencers that depict positive alcohol-related outcomes.

In our research, we analyzed popular bartenders’ content videos disseminated on TikTok©. Bartenders are defined as people who “give instructions on how to make alcoholic drinks like cocktails” [18]. They are trendy, with, for instance, the most popular bartender on TikTok©, “@realtipsybartender,” reaching 8.9 million followers in April 2023. His cocktail recipe, “Candy Unicorn Gin & Tonic,” generated 1.9 million ‘likes’.

This study aimed to characterize the video content posted by influential bartenders on TikTok**©**. Investigating the portrayal of alcohol on emerging social media platforms such as TikTok**©** is important. Our results can provide valuable insights into how alcohol is promoted through previously unexplored channels and provide valuable context into how online content can translate into real-life drinking behaviours. The findings also provide valuable information for regulatory agencies and policymakers to understand how alcohol is being promoted on social media platforms frequented by young people and others, and how digital marketing can be regulated.

## Methods

### Data collection

A multi-step process was undertaken to identify the top global bartender influencers on TikTok**©**. We (EG and MZ) created a DataMiner to collect the unique URLs of videos under the #bartender hashtag from TikTok**©**s’ desktop version, identifying 978 videos. We also collected the unique creator profile URLs of accounts posting at least one video with the #bartender hashtag. Next, EG and MZ created a second scraper to retrieve the profile information from each account posting at least one video under the #bartender hashtag (n = 295). EG then visited the unique profile URL of each account to determine if their account was dedicated to bartending. We considered an account dedicated to bartending if it met two criteria: (1) the content was mainly focused on recipes and/or drink making; (2) the account was in English. Eighty-six accounts met the two criteria (32% of the 295 accounts).

To identify other popular bartenders who could have been missed from our sampling process or did not post videos with the hashtag #bartender, EG completed Google searches with keywords attempting to identify more dedicated bartending accounts. Thirteen new accounts met the inclusion criteria (total, 99). Here, we set a criterion to only include the most popular bartending accounts, defined as accounts with 1 + million followers (arbitrary cut-off for this exploratory study), leaving a final sample of 15 bartending accounts for further data collection.

After sampling the accounts of bartenders included in our study, we focused on a narrower period, given our time and resource constraints. We scraped TikTok**©** content of these bartenders for November and December 2021, the period just before Christmas and New Year, when alcohol tends to be more marketed than in other periods [19]. We collected the visual content and metadata of 732 videos. We manually downloaded each video and assigned it a unique identification number corresponding to an Excel spreadsheet. EG viewed each video to determine whether the video was giving information on how to make an alcoholic beverage. In total, 345 videos (out of the 732) were included for analysis to understand characterizing patterns. The study did not require ethics approval because all data was posted publicly and shared without expectation of privacy.

### Coding

The videos were qualitatively analyzed by EG, following the content analysis method used by other social media studies [15, 20, 21] to identify the elements described below: (1) presence of alcohol, including through the display of a brand or of a bottle or other container that is used for alcohol; (2) alcohol categories: vodka, whisky, liquors (spirit drink produced by flavouring an alcoholic base with fruits, plants, eggs or dairy products by different processes such as maceration or infusion and by adding sugar), ‘unspecified’ when it was not possible to identify categories; (3) alcohol brand(s) (if visible); (4) presence of sweet products: candy, chocolate and ice cream as ingredients of cocktails combined with alcohol, and their brand names (if visible); and (5) cues that could be appealing to young people: video games, references to cartoons or movies aimed at young people.

We monitored the presence of sweet products because previous studies have noted that flavoured alcoholic beverages are marketing tools that target young people [22]. In the tobacco context, candy-like flavoured cigarettes were launched to decrease harm perceptions and encourage young people to smoke [23]. Consequently, there is the risk that combining candies with alcohol in popular cocktails produces similar outcomes.

We included “mocktails” (cocktails without alcohol) in our study as alcohol companies could use them as a brand diversification strategy.

To develop the coding frame, each author independently reviewed videos until observation saturation. The authors met, compared observations, and co-created a coding frame, which was then test-coded by EG and MZ. After minor modifications, the final code frame was approved by all authors. Following EG’s coding of each video, we used descriptive statistics to identify the most frequently displayed alcohol categories, brands, and other elements described above. The cumulative views were also calculated to get information on the dissemination of the analyzed videos.

## Results

The 345 analyzed videos received 270,325,600 views (average, 783,552 per video) (Table 1, C6), 28,488,195 ‘likes’ (average, 82,574 per video), 189,105 comments (average, 548 per video) and 562,038 shares (average, 1,629 per video). On average, creators had 2 M followers. The most popular account in our sample was @realtipsybartender (4.9 M followers), followed by @michellebellexo (2.9 M) and @theparadisebartender / @drinkowithrico (2.6 M each) (Table 1, C1).

**Table 1:** – Overview of TikTok’s Top-Viewed Bartender Influencer Accounts

Bartender Name	Number of followers on TikTok^©^ (C1)	Frequency of alcohol brand appearances in the videos analyzed (C2)	# of sweetened products appearances in the videos analyzed (C3)	# of young people cues in the videos analyzed (C4)	# of views of the videos analyzed (C6)
@realtipsybartender	4.9 M	123	23	5	89,258,500
@michellebellexo	2.9 M	12	2		22,113,500
@theparadise.bartender	2.6 M	23	1	2	1,292,000
@drinkowithrico	2.6 M	24	2	1	15,447,500
@johnrondi	2.3 M	24	2		30,910,900
@timthetank	2.1 M	42	11	3	8,954,400
@bartenderis	2.1 M				74,700
@barchemistry	1.8 M	10	1	1	2,893,200
@barrisstaelba	1.4 M				823,800
@courtneyshae_	1.3 M	6			14,202,000
@nic.hamilton	1.3 M	28			34,029,200
@sincitybartender	1.3 M	12	7	29	16,547,100
@lasvegas.bartender	1.1 M	18	4	1	5,218,000
@cdbartending	1 M	22			1,422,500
@thewhyteelephant	1 M	21	2	1	27,138,300
					**270,325,600**

Ninety-two per cent of videos displayed alcohol (317 videos; 249,275,600 views), and 8% were mocktails (28 videos; 21,050,000 views). Of the 317 videos featuring alcohol, the most frequent categories were liquor (161 videos; 51% of videos with alcohol), vodka (117; 37%), rum (68; 22%), whisky (66; 21%), tequila (29; 9%) and gin (21; 7%) (some videos could contain multiple categories). Other alcohols were marginally present, such as wine, champagne or beer.

Of the 317 videos with alcohol, 230 featured brands (73%, 207,187,700 views) (Table 1, C2). The most present brands were “Tipsy Bartender” affixed on bottles (not as an alcohol brand but as a celebrity influencer brand) (30 videos; 9%; 9,603,200 views), the vodka brand “Smirnoff” (20; 6%; 18,755,700 views), and the liquor “DeKuyper” (17; 5%; 10,514,800). The brand whisky “Hiram Walker” has the highest peak of views (30,989,500), followed by “Barefoot” wine (27,657,300).

19% of videos (n = 67) used sweet products in the alcohol and non-alcohol cocktail recipes (total, 68,378,800 views): candies, chocolate, ice cream or sweets-flavored (Table 1, C3). Of the 67 videos, 55 combined sweets with alcohol ingredients (82%; 53,957,900 views with an average of 981,053 views per video; 17% of the 317 alcohol videos). Candies were the most observed sweet product (38 videos; 68%; 46,014,600 views), followed by ice cream (13 videos; 23%; 7,731,200 views) and chocolate (5 videos; 9%; 7,948,400 views). Four recipes with alcohol/sweets claimed to imitate the taste of certain sweets or highlight a particular soda (i.e., the same taste as a specific soda or the same taste as a specific candy) (2,436,700 views), and some featured several sweets within the same video. Out of the 55 videos with sweet products and alcohol, 18 (33%) displayed a sweet brand or a reference to a brand (e.g. a cocktail that tastes like a mentioned brand soda/candy) (Table 1, C5). The most cited brands were “Swedish Fish” candy (5 videos; 9%, 1,253,300 views) and “Skittles” candy (4; 6%; 1,163,900 views).

Last, 13% (n = 43) presented a recipe with alcohol-containing cues to young people’s interests (15,763,300 views, with an average of 366,588 views per video). For instance, some refer to “Harry Potter’s” butterbeer, to the Christmas movie “The Grinch,” or to the “Sonic” video games (Table 1, C4 and C5).

## Discussion

This study is the first to analyze the content of videos posted by popular bartenders (between 1 and 4.9 million followers) on TikTok**©**. Our paper revealed that in a two-month period around Christmas, when alcohol is usually aggressively marketed, 92% of the 345 videos posted on TikTok**©** included visuals of alcohol, receiving 250 M + views, with an average of 786,358 views per video. Content promoted high-ABV beverages and alcohol brands, which were visible in 73% of the videos. 19% of videos combined alcohol and sweet products (i.e. candies) in their recipes, and 12% contained cues with suggestive appeals to young people.

The first finding is that a large percentage of videos analyzed (92%) included alcohol in recipes. Considering that the videos had millions of views, there is a high risk that users on TikTok©, including young people and minors, are exposed to bartenders’ alcohol posts. In addition, previous research has shown easy access to social media platforms in general, whatever the age, even under 13, the minimum age to “legally” use TikTok©, despite these legal age restrictions [24]. This stresses the importance of addressing the fact that popular bartenders’ alcohol videos are easily accessible to young people and may appear on their algorithm-curated TikTok For You Page.

This research reveals the presence of alcohol brands in a high percentage of the analyzed videos (73%), showing that TikTok©’s rules are insufficient: “*You must not post Branded Content which promotes products or services from the following prohibited industries: alcohol - alcoholic beverages (wine, beer, spirits, etc.), alcohol clubs/subscription services, alcohol-making kits, or alcohol-sponsored events*.^1^ Proving the commercial link between the bartenders and the brands is difficult. Recently, the United States Federal Trade Commission sent warning letters to two trade groups – the American Beverage Association and The Canadian Sugar Institute for hiring dietician influencers to promote the safety of their products without declaring their financial relationship [25]. Given the difficulties of regulating cross-border marketing [26], this is a particularly difficult challenge. Indeed, the online environment enabled by TikTok content is not confined to specific geographic locations and is spread across countries. Both users and influencers operate across different countries. It is not feasible for regulatory entities to identify who saw the promotional materials of users in their area if the influencer is operating in another country. Regulatory entities are limited in their ability to monitor and enforce disclosure laws due to the difficulties in identifying influencer marketing, data availability, and monitoring capabilities. Nevertheless, alcohol exposure, whether paid or not by alcohol companies, influences social media users. Self-regulation of such digital platforms has thus limitations. Going further, and in the French context, where three of the researchers for the present study were based, the Evin law, which restricts the marketing of alcohol, in particular to young people, could be inspiring for other countries. This law applies to any drinks over 1.2% alcohol by volume and regulates direct and indirect alcohol media advertising [27]. Regarding digital media (websites, social media, apps), alcohol ads are allowed except when they are displayed on platforms and websites that target young people and if they are intrusive (e.g. “pop-up” ads). In authorized situations, ads must only contain factual/informative data and objective qualities on alcohol (e.g., proof, origin, composition and means of production), and must display the warning ‘alcohol abuse is dangerous for health’ [28]. In France, the bartenders’ videos we analyzed with visible alcohol brands do not respect French law. A similar situation was brought to Court in January 2023, with 37 posts published on Instagram© by popular influencers improperly promoted alcohol brands and had to be removed by Meta (owner of Facebook and other social media platforms), following a lawsuit from a civil society organization^2^. The non-respect of the Evin law raises the question of implementing stricter (and thus clearer) regulations of comprehensive advertising ban, including advertising on social media, similar to bans introduced in Norway in 1975 [29] or Lithuania in 2017 [30]. Norway’s ban covers all media, with a few exceptions to advertising in trade magazines and other platforms, and evidence shows that the ban led to a lasting reduction in alcohol sales [31]. In Lithuania, some exemptions exist in the recently introduced alcohol advertising ban, such as the possibility of having brand logos at alcohol point-of-sale and information shared by specialists. A recent study revealed high compliance with Lithuania’s alcohol advertising ban on such platforms [32].

Our study has limitations. First, a relatively “small” sample of videos was analyzed: we only studied TikTok© as a key social media, focused on bartenders only, and videos posted during a two-month period around Christmas. Platforms and popular influencers are in constant evolution; future investigations should be carried out during longer periods to follow online trends. A second limitation is that we looked at the content of the bartenders’ videos, but we did not explore the reactions of users, particularly young people, to these posts (attitudes, alcohol perception, drinking motivation after watching them). Our study was exploratory; we used the #bartender hashtag only, but we recognized that bartenders might use others. In addition, we noted that some of the videos might be appealing to young people, but this would merit further investigations, and we are not in a position to prove that the videos were targeting that specific audience. Moreover, we did not specifically examine TikTok’s policies or marketing. Future research could focus on how TikTok’s algorithm may promote alcohol and other products on the For You Page (FYP) and/or be marketed against the platform’s terms of service.

Finally, our results encourage public health actors to consider working on platforms widely used by the public and young people to prevent alcohol-related risks and, at the same time, to counter the presence of attractive influencers’ alcohol posts.

## Data Availability

The datasets used and/or analyzed during the current study are available from the corresponding author upon reasonable request.

## Footnotes

1 https://www.tiktok.com/legal/page/global/bc-policy/en.

2 https://movendi.ngo/news/2023/02/07/addictions-france-wins-lawsuit-against-meta-over-alcohol-ads/.
